# Comparative Effectiveness Research: An Empirical Study of Trials Registered in ClinicalTrials.gov

**DOI:** 10.1371/journal.pone.0028820

**Published:** 2012-01-09

**Authors:** Florence T. Bourgeois, Srinivas Murthy, Kenneth D. Mandl

**Affiliations:** 1 Division of Emergency Medicine, Children's Hospital Boston, Boston, Massachusetts, United States of America; 2 Department of Pediatrics, Harvard Medical School, Boston, Massachusetts, United States of America; 3 Department of Pediatrics, Hospital for Sick Children, Toronto, Canada; 4 Children's Hospital Informatics Program at the Harvard-MIT Division of Health Sciences and Technology, Children's Hospital Boston, Boston, Massachusetts, United States of America; University of Louisville, United States of America

## Abstract

**Background:**

The $1.1 billion investment in comparative effectiveness research will reshape the evidence-base supporting decisions about treatment effectiveness, safety, and cost. Defining the current prevalence and characteristics of comparative effectiveness (CE) research will enable future assessments of the impact of this program.

**Methods:**

We conducted an observational study of clinical trials addressing priority research topics defined by the Institute of Medicine and conducted in the US between 2007 and 2010. Trials were identified in ClinicalTrials.gov. Main outcome measures were the prevalence of comparative effectiveness research, nature of comparators selected, funding sources, and impact of these factors on results.

**Results:**

231 (22.3%; 95% CI 19.8%–24.9%) studies were CE studies and 804 (77.7%; 95% CI, 75.1%–80.2%) were non-CE studies, with 379 (36.6%; 95% CI, 33.7%–39.6%) employing a placebo control and 425 (41.1%; 95% CI, 38.1%–44.1%) no control. The most common treatments examined in CE studies were drug interventions (37.2%), behavioral interventions (28.6%), and procedures (15.6%). Study findings were favorable for the experimental treatment in 34.8% of CE studies and greater than twice as many (78.6%) non-CE studies (P<0.001). CE studies were more likely to receive government funding (P = 0.003) and less likely to receive industry funding (P = 0.01), with 71.8% of CE studies primarily funded by a noncommercial source. The types of interventions studied differed based on funding source, with 95.4% of industry trials studying a drug or device. In addition, industry-funded CE studies were associated with the fewest pediatric subjects (P<0.001), the largest anticipated sample size (P<0.001), and the shortest study duration (P<0.001).

**Conclusions:**

In this sample of studies examining high priority areas for CE research, less than a quarter are CE studies and the majority is supported by government and nonprofits. The low prevalence of CE research exists across CE studies with a broad array of interventions and characteristics.

## Introduction

Comparative effectiveness (CE) research is the “generation and synthesis of evidence that compares the benefits and harms of alternative methods to prevent, diagnose, treat and monitor health conditions in ‘real world’ settings”.[1] Recognizing that the evidence-base for the practice of medicine is often built on studies lacking active comparators and therefore falls short in supporting either high quality care or healthcare reform, there is now substantial focus on and investment in CE research.[2] In the United Kingdom, for instance, the National Institute for Health and Clinical Excellence compiles and disseminates CE and cost-effectiveness data to support diagnostic and therapeutic decisions.[3], [4] Similar agencies in Canada and Australia—the Common Drug Review and the Pharmaceutical Benefits Advisory Committee, respectively—provide information on the effectiveness and cost of pharmaceuticals, specifically, compared to relevant alternatives.[5], [6], [7]

In the United States, CE research was recently appropriated $1.1 billion through the American Recovery and Reinvestment Act of 2009.[1], [8] This funding reflects the growing awareness that improved data is needed on the relative benefits of therapies to enable patients and clinicians to make informed decisions and to reduce gross geographic variations in healthcare allocations seen across the United States.[9], [10]

In order to envision how the evolution of CE research will shape the evidence-base for future healthcare delivery, we sought to leverage a novel data source of clinical trials—the web-based registry ClinicalTrials.gov—and measure the prevalence of CE research and characterize current CE research activity. We focus our empirical study on research conducted in the United States where the concerted effort to expand CE research has not yet had a substantial impact on studies performed. Specifically, we examine research areas highlighted in the 2009 Institute of Medicine (IOM) list of 100 priority topics deemed to be most pertinent to improving the health of the population, commissioned by the United States Congress to inform the initial investment in CE research.[11] Since we focus on research activity in the United States, we limit our study to trials registered in ClinicalTrials.gov, which is the primary registry employed by investigators in the United States and which has previously been used to define and study large trial cohorts.[12], [13] To begin to anticipate the impact of the investment in CE research, we determine the prevalence of CE research to date, the types of interventions studied, and the role of funding sources sponsoring CE research.

## Methods

### Selection of Clinical Studies

**Figure 1:**
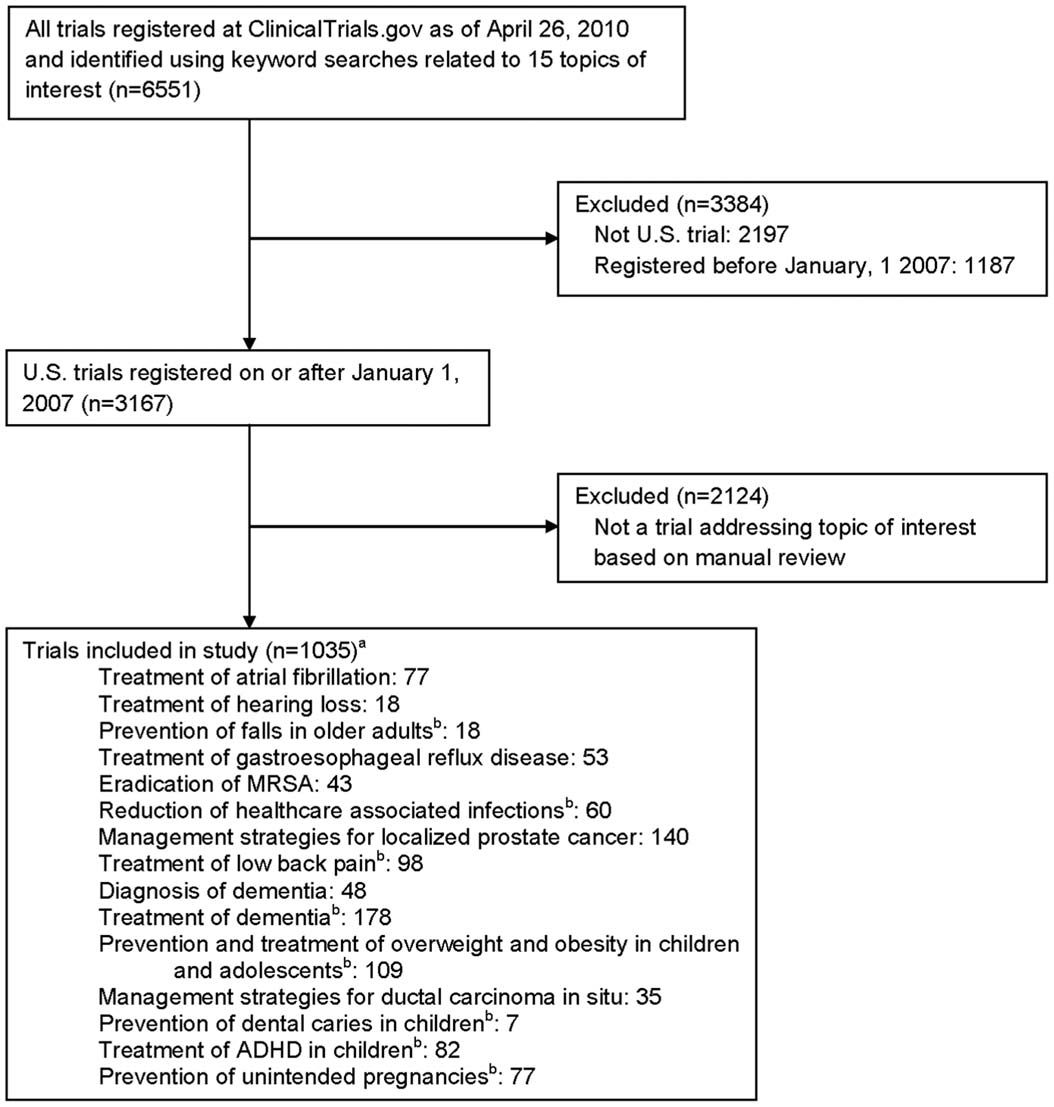
Study Flow Diagram. Selection of trials in ClinicalTrials.gov that address 15 research topics identified by the Institute of Medicine as being top priority for comparative effectiveness research.

We examined the 15 research areas among the top 25 topics on the IOM list of priority areas that addressed specific diseases or conditions as opposed to strategies for delivering care or diagnostic and treatment approaches for broad groups of conditions (Table S1).[11] We identified studies pertaining to these research areas in ClinicalTrials.gov, selecting trials that were registered between January 1, 2007 and April 26, 2010 (date of data download from ClinicalTrials.gov) and that were conducted in the United States (Figure 1).

ClinicalTrials.gov is a web-based registry of clinical studies that provides a publicly available source of information on clinical studies conducted in the United States and internationally.[14] In 2005, the International Committee of Medical Journal Editors instituted a policy requiring prospective registration of all trials—regardless of intervention type—as a prerequisite for publication, resulting in a dramatic increase in the registration of trials and sustained wide-spread use since then.[15], [16] In addition, under the FDA Amendments Act of 2007, the FDA requires the registration in ClincialTrials.gov of all clinical investigations (except phase I trials) of a drug, biologic, or device that is subject to FDA regulation, regardless of trial design.[17] Users can query the registry and identify specific types of trials using a search function that includes keyword searches. We employed keywords identified from published systematic reviews on the diseases or conditions of interest (Table S1). Studies selected using this search strategy were individually reviewed and those included that directly addressed the research topics of interest.

### Definitions and Data Extraction

CE studies were defined as those comparing the experimental intervention to another active therapy as opposed to a placebo control or no comparator.[18] Both the experimental treatment and the comparator were classified as an intervention involving a drug, device, procedure, behavioral change, or other treatment (e.g. dietary supplement). Active comparator studies were defined as studies that compared two treatment alternatives, including “optimal usual care” when these reflected appropriate current practice and standards.[2], [11] In determining the type of comparator employed, we did not rely on the investigator-assigned study labels in ClinicalTrials.gov but rather examined the detailed description of the study in the record.

The data elements obtained from the ClincialTrials.gov entry and recorded for each study were registration date, study start and completion dates, experimental treatment under study, comparator type, trial phase for drug and device studies, funding source, outcome measures, anticipated enrollment number, subject age groups, and elements of the study design.

Study outcome measures are specifically listed in the study record as primary and secondary outcomes and we determined whether these included measures of safety, including any side effects, adverse events, or other potential harms or risks related to the intervention, or cost assessments, including formal cost-analyses and general measures of resource utilization. For 17 studies that did not include specific outcome measures in the study record, we reviewed the study descriptions to identify the inclusion of safety and cost assessments.

Funding source was classified as government, industry, or nonprofit based on the funding sponsors listed in the record. We categorized “NIH”, “U.S. federal”, and “other government” as government funding; “industry” as industry funding; and “network”, “individual”, and “other” (which includes universities, hospitals, foundations, and other nonprofit organizations) as nonprofit funding.[12] We applied this classification to primary and secondary funding sources. Subject ages are categorized in the registry as “Child” (up to 17 years), “Adult” (18 to 65 years), “Senior” (66 years and older), and combinations of these groups. We re-coded these data into a three-level variable of children only, children and adults/seniors, and adults/seniors.

Classification of experimental intervention, comparator type, and safety and cost assessments were performed independently by two of the authors (F.B. and S.M.) and disagreements resolved by consensus.

### Assessment of Study Outcomes

Publications associated with studies were identified using a previously described method.[12] Briefly, for studies that did not include results or a reference to a publication within the CinicalTrials.gov record, four electronic databases were searched. These included PubMed, the Cochrane Library, EMBASE, and the Cumulative Index to Nursing and Allied Health Literature (CINAHL). All searches were finalized by August 31, 2010. Each publication was reviewed and the results for the primary outcome classified as favorable (i.e. statistically significant based on P values or confidence intervals) or not favorable (i.e. not statistically significant) for the experimental treatment. For studies without a comparator or statistical analysis, the classification was based on the interpretation of the results provided in the study conclusions. Publications that did not describe results pertaining to the efficacy or safety of the intervention were classified as “neither”. Two of the authors (F.B. and S.M.) independently performed the outcome classification and resolved disagreements by consensus. Inter-coder agreement for assigning study outcomes was good with a kappa of 0.78 (95% CI, 0.65–0.91).

### Statistical Analysis

We calculated the proportion of studies that were CE studies and compared study characteristics for CE and non-CE studies. Sub-analyses were performed on CE studies based on funding source. Trials examining a pharmaceutical intervention were also specifically examined and CE and non-CE studies compared. Chi-square and Kruskal-Wallis tests were used to compare categorical and median values, respectively. We used the Cochran-Mantel-Haenszel test to control for funding source when examining study outcomes. All data were analyzed with SAS software (version 9.2, SAS Institute Inc., Cary, North Carolina).

## Results

Of the 3167 studies retrieved from ClinicalTrials.gov, 1035 were included in the final study sample after reviewing the study description (Figure 1). Among these, 231 (22.3%; 95% confidence interval [CI], 19.8%–24.9%) were CE studies and 804 (77.7%; 95% CI, 75.1%–80.2%) were non-CE studies, with 379 (36.6%; 95% CI, 33.7%–39.6%) employing a placebo control and 425 (41.1%; 95% CI, 38.1%–44.1%) no control.

Study characteristics are presented in Table 1. In half the studies examined (49.9%), the experimental treatment consisted of a pharmacological therapy and in 18.3% a behavioral intervention. The distribution of experimental treatments differed for the different study types, with drug treatments more likely to be studied with a placebo or no intervention (P<0.001). Studies with active comparators were more likely to be in advanced phases (Phase 3 or 4; P<0.001), to employ larger sample sizes (P<0.001), and to be longer in duration (P = 0.02). Fewer studies with active comparators included a primary safety outcome (8.2% vs. 14.0% and 23.8% for placebo-controlled and no comparator studies; P<0.001) and only 3.5% included a cost assessment.

**Table 1:** Comparative Effectiveness Studies Registered in ClinicalTrials.gov.

Characteristic	Category	Total (n = 1035)	Study Type		
			Comparative Effectiveness Study: Active Comparator (n = 231)	Non-Comparative Effectiveness Study: Placebo Control (n = 379)	Non-Comparative Effectiveness Study: No Control (n = 425)
**Experimental treatment, n (%)** a	**Drug**	516 (49.9)	86 (37.2)	226 (59.6)	204 (48.0)
	**Device**	121 (11.7)	32 (13.8)	27 (7.1)	62 (14.6)
	**Procedure**	146 (14.1)	36 (15.6)	22 (5.8)	87 (20.5)
	**Behavioral change**	189 (18.3)	66 (28.6)	77 (20.3)	46 (10.8)
	**Other**	48 (4.8)	11 (4.8)	26 (6.9)	11 (2.6)
	**None**	15 (1.4)	0	0	15 (3.5)
**Study phase, n (%)** b, c	**Phase 1, 2, 2/3**	275 (43.2)	40 (33.9)	105 (41.2)	131 (49.2)
	**Phase 3, 4**	230 (36.1)	52 (44.1)	101 (39.6)	78 (29.3)
	**Unknown**	132 (20.7)	26 (22.0)	49 (19.2)	57 (21.4)
**Primary safety outcome, n (%)** a	**Yes**	173 (16.7)	19 (8.2)	53 (14.0)	101 (23.8)
**Cost assessment, n (%)**	**Yes**	24 (2.3)	8 (3.5)	11 (2.9)	5 (1.2)
**Anticipated sample size, median (IQR Q1,Q3)** a,e		100 (40, 280)	160 (78, 350)	147 (56, 327)	60 (30, 164)
**Age of study population, n (%)** a	**Children only**	171 (16.5)	40 (17.3)	50 (11.8)	81 (21.4)
	**Children and adults**	124 (12.0)	29 (12.6)	42 (9.9)	53 (14.0)
	**Adults only**	740 (71.5)	162 (70.1)	333 (78.4)	245 (64.6)
**Length of study, median years (IQR Q1,Q3)** c, f		2.1 (1.1, 3.3)	2.4 (1.4, 3.7)	2.0 (1.0, 3.2)	2.0 (1.0, 3.2)

Abbreviations: IQR, interquartile range.

a P<0.001 for chi-square and Kruskal-Wallis tests for categorical and median values, respectively.

b Phase data applies to 637 drug and device trials.

c P = 0.02 for chi-square.

d Randomization applies to 610 trails with an active comparator or placebo control.

e Sample size data available for 1025 trials.

f Study length available for 860 trials.

### Impact of Funding Source on Characteristics of CE Studies

The distribution of primary funding sources was similar among CE and non-CE studies (Table 2). Overall, 71.8% (n = 166) of CE studies were funded by non-commercial sources, including government and nonprofit organizations. CE studies were more likely to include government funding (32.5% compared with 22.9% of non-CE studies, P = 0.003) and less likely to include industry funding (37.2% compared with 46.4% of non-CE studies, P = 0.01).

**Table 2:** Funding Sources for Comparative Effectiveness and Non-Comparative Effectiveness Studies Registered in ClinicalTrials.gov.

Characteristic	Category	Total (n = 1035), n (%)	Study Type		P-value
			Comparative Effectiveness Study (n = 231), n (%)	Non-Comparative Effectiveness Study (n = 804), n (%)	
**Primary funding source**	**Government**	110 (10.6)	32 (13.8)	78 (9.7)	0.10
	**Industry**	334 (32.3)	65 (28.1)	269 (33.5)	
	**Nonprofit**	591 (57.1)	134 (58.0)	458 (57.0)	
**Government funding**	**All or some government funding**	259 (25.0)	75 (32.5)	184 (22.9)	0.003
	**No government funding**	776 (75.0)	156 (67.5)	620 (77.1)	
**Industry funding**	**All or some industry funding**	459 (44.4)	86 (37.2)	373 (46.4)	0.01
	**No industry funding**	576 (55.6)	145 (62.8)	431 (53.6)	

We further examined study interventions and other characteristics for CE studies based on funding source (Table 3). Among CE studies funded primarily by industry, 95.4% involved the study of a drug or device and most compared the intervention to another drug or device (90.8%). Primarily industry-funded CE studies involved the largest anticipated sample size (median of 324 subjects vs. 175 and 100 subjects for government and nonprofit funding, respectively; P<0.001), were the least likely to enroll pediatric subjects (7.6% vs. 37.5% and 17.3% for government and nonprofit funding, respectively; P<0.001), and were the shortest in duration (median length 1.8 years vs. 3.0 and 2.4 years for government and nonprofit funding, respectively; P<0.001).

**Table 3:** Characteristics of Comparative Effectiveness Studies by Funding Source.

Characteristic	Category	Total (n = 231)	Primary Funding Source				Government Funding		
			Government (n = 32)	Industry (n = 65)	Nonprofit (n = 134)	P-value	All or Some Government (n = 75)	No Government (n = 156)	P -value
**Experimental treatment, n (%)**	**Drug**	86 (37.2)	10 (31.2)	45 (69.2)	31 (23.1)	<0.001	19 (25.3)	67 (43.0)	<0.001
	**Device**	32 (13.8)	2 (6.2)	17 (26.2)	13 (9.7)		3 (4.0)	29 (18.6)	
	**Procedure**	36 (15.6)	1 (3.1)	2 (3.1)	33 (24.6)		9 (12.2)	27 (17.3)	
	**Behavioral change**	66 (28.6)	18 (56.2)	1 (1.5)	47 (35.1)		40 (53.3)	26 (16.7)	
	**Other**	11 (4.8)	1 (3.1)	0	10 (7.5)		4 (5.3)	7 (4.5)	
**Comparison type, n (%)**	**Drug vs. drug**	76 (32.9)	8 (25.0)	46 (70.8)	22 (16.4)	<0.001	14 (18.7)	62 (39.7)	<0.001
	**Device vs. device**	27 (11.7)	2 (6.2)	13 (20.0)	12 (9.0)		3 (4.0)	24 (15.4)	
	**Procedure vs. procedure**	29 (12.6)	1 (3.1)	1 (1.5)	27 (20.2)		7 (9.3)	22 (14.1)	
	**Behavioral change vs. behavioral change**	64 (27.7)	17 (53.1)	0	47 (35.1)		39 (52.0)	25 (16.0)	
	**Other**	35 (15.1)	4 (12.5)	5 (7.7)	26 (19.4)		12 (16.0)	23 (14.8)	
**Primary safety outcome, n (%)**	**Yes**	18 (7.8)	1 (3.1)	14 (21.5)	4 (3.0)	<0.001	2 (2.7)	17 (10.9)	0.03
**Cost assessment, n (%)**	**Yes**	8 (3.5)	0	1 (1.5)	7 (5.2)	0.21	2 (2.7)	6 (3.8)	0.65
**Anticipated sample size, median (IQR Q1,Q3)** a		160 (80, 355)	175 (121, 288)	312 (205, 550)	100 (57, 240)	<0.001	160 (80, 300)	155 (61, 400)	0.65
**Age of study population, n (%)**	**Children only**	40 (17.3)	12 (37.5)	5 (7.7)	23 (17.2)	<0.001	22 (29.7)	18 (11.5)	<0.001
	**Children and adults**	29 (12.6)	5 (15.6)	3 (4.6)	21 (15.7)		12 (16.2)	17 (10.9)	
	**Adults only**	162 (70.1)	15 (46.9)	57 (87.7)	90 (67.2)		41 (54.7)	121 (77.6)	
**Length of study, median years (IQR Q1,Q3)** b		2.4 (1.3, 3.7)	3.0 (2.4, 4.2)	1.9 (1.1, 2.7)	2.4 (1.7, 3.9)	<0.001	2.9 (2.2, 4.2)	2.2 (1.2, 3.0)	<0.001

a Sample size data available for 229 trials.

b Study length available for 199 trials.

Studies with any type of government funding were less likely to study a drug or device (P<0.001) and more likely to include children (P<0.001) and be longer in duration (P<0.001). Government funding was not associated with an increase in the study of safety or cost outcomes.

### Pharmaceutical and Device Studies and CE Research

Among the subset of 516 studies examining a pharmaceutical intervention, 86 (16.7%; 95% CI, 13.4%–19.9%) were CE studies and 430 (83.3%; 95% CI, 80.1%–86.6%) were non-CE studies.

Government sources provided primary funding for 11.6% (n = 10) and industry for 52.3% (n = 45) of CE drug studies (Table 4). CE studies were less likely to include a safety outcome compared with non-CE studies (P<0.001), involved larger anticipated sample sizes (median of 238 subjects vs. 80 subjects; P<0.001), and were less likely to be double-blinded (66.7% vs. 92.7%; P<0.001). Device studies included 32 (26.4%; 95% CI, 18.6%–34.3%) CE studies and 89 (73.6%; 95% CI, 65.7%–81.4%) non-CE studies. Government was the primary funding source for 6.2% (n = 2) and industry 53.1% (n = 17) of the CE device studies.

**Table 4:** Study Characteristics of Drug Trials Registered in ClinicalTrials.gov.

Characteristic	Category	Total (n = 516)	Trial Type		P-value
			Comparative Effectiveness Trial (n = 86)	Non-Comparative Effectiveness Trial (n = 430)	
**Primary funding source, n (%)**	**Government**	36 (7.0)	10 (11.6)	26 (6.0)	0.11
	**Industry**	260 (50.4)	45 (52.3)	215 (50.0)	
	**Nonprofit**	220 (42.6)	31 (36.0)	189 (44.0)	
**Study phase, n (%)**	**Phase 1, 2, 2/3**	247 (47.9)	37 (43.0)	210 (48.8)	0.58
	**Phase 3, 4**	193 (37.4)	36 (41.9)	157 (36.5)	
	**Unknown**	76 (14.7)	13 (15.1)	63 (14.6)	
**Primary safety outcome, n (%)**	**Yes**	119 (23.1)	7 (8.1)	112 (26.0)	<0.001
**Cost assessment, n (%)**	**Yes**	12 (2.3)	3 (3.5)	9 (2.1)	0.43
**Study design: observational vs. interventional, n (%)**	**Observational**	10 (1.9)	2 (2.3)	8 (1.9)	0.78
**Randomization, n (%)** a	**Yes**	297(95.2)	81 (94.2)	216 (95.6)	0.61
**Blinding, n (%)** b	**Double-blind**	256 (86.2)	54 (66.7)	202 (92.7)	<0.001
	**Single-blind**	9 (3.0)	6 (7.4)	3 (1.4)	
	**No blinding**	32 (10.8)	21 (25.9)	11 (5.1)	
**Anticipated sample size, median (IQR Q1,Q3)** c		100 (36, 292)	238 (90, 508)	80 (30, 255)	<0.001
**Age of study population, n (%)**	**Children only**	83 (16.1)	12 (14.0)	71 (16.5)	0.71
	**Children and adults**	34 (6.6)	7 (8.1)	27 (6.3)	
	**Adults only**	399 (77.3)	67 (77.9)	332 (77.2)	
**Length of study, median years (IQR Q1,Q3)** d		1.6 (0.9, 2.9)	2.0 (1.1, 2.7)	1.6 (0.8, 2.9)	0.30

Abbreviations: IQR, interquartile range.

a Applies to 312 trials with an active or placebo control.

b Applies to 297 trials that were randomized.

c Sample size data available for 511 trials.

d Study length available for 423 trials.

### CE Study Outcomes

Results were identified for 115 (11.1%) studies. A total of 8/23 (34.8%) reports described positive findings for studies with active controls compared with 66/84 (78.9%) among non-CE studies (12/32 [73.9%] placebo-controlled trials and 6/32 [84.2%] trials without controls) (P<0.001). Among trials primarily funded by industry, 33/41 (80.5%) reported positive findings compared with 41/66 (61.7%) among all others (P = 0.04). After controlling for primary funding source, CE studies remained less likely to report positive findings (P<0.007 for Cochran-Mantel-Haenszel test). Among CE studies involving a drug therapy, findings were positive for 30.0% (n = 3) of CE studies compared with 81.6% (n = 40) of non-CE studies (P<0.001, Cochran-Mantel-Haenszel test controlling for primary funding source). None of the CE studies examining devices and 71.4% (n = 5) of non-CE studies involving devices reported findings favorable for the device (P = 0.04).

## Discussion

We provide a benchmark for the current state of CE research, demonstrating that for conditions deemed as highest priority by the IOM, less than a quarter of studies examined comparative effectiveness. The majority of CE studies were funded by government and nonprofit sources and outcomes were less likely to be positive for the experimental intervention among CE trials compared with non-CE trials. Funding sources had a substantial impact on the characteristics of CE studies, with industry-funded trials focusing primarily on drugs and devices and those funded by noncommercial sources addressing more diverse types of interventions. Industry-funded trials also differed in trial design with larger sample sizes, fewer studies involving pediatric patients, and shorter study periods.

Only a small proportion of CE studies address safety and cost outcomes, highlighting an opportunity for government-sponsored CE research to play a significant role. [2], [19], [20], [21] Regardless of funding source, CE studies are less likely to examine safety outcomes, particularly among drug studies, demonstrating an emphasis on measuring treatment efficacy over measuring treatment risks and adverse events. Cost assessments are currently rare for both CE and non-CE studies across all funding sources.

Hochman et al previously found low prevalence of CE studies and a higher rate of positive outcomes among non-CE studies of pharmaceuticals compared with CE studies. [18] Using a comprehensive and growing data source of recent and ongoing research activity, our results corroborate those findings. We further demonstrate that the low prevalence of CE research and differences in outcomes exist across CE studies with a broad array of interventions and that characteristics of CE studies vary substantially based on the funding source sponsoring the study.

With an ever-expanding list of diagnostic and therapeutic options, CE studies fill an important gap in informing clinicians whether an intervention is superior to existing and familiar alternatives. Our findings suggest characteristics of CE research that may produce specific shifts in the evidence-base towards more critical and comprehensive assessments of the intervention under study. From our findings, we extrapolate that the projected increase in the number of CE studies—particularly studies of drugs and devices funded by noncommercial sources—will increase the proportion of studies that fail to support adoption of the experimental treatment. We base this prediction on two findings.

The first is that CE studies are less likely than non-CE studies to report results that promote the use of the experimental intervention, reinforcing that this study design may produce more conservative results in terms of the superiority of a therapy compared to other treatments. Trials with inactive comparators have previously been shown to have a greater likelihood of achieving favorable findings.[18], [22], [23] Drug and device studies that employ non-active comparators and yield favorable outcomes may encourage the adoption and use of the experimental intervention even though information is lacking on how the drug or device compares to current standards of care.[23]

Secondly, while noncommercial sources funded 71% of CE studies overall, industry funded the majority of CE drug and device studies, which biases toward results supporting the use of a product.[24], [25], [26], [27] Industry trials investigating drugs and devices are typically designed and conducted by the company marketing the product and there is substantial and well-documented evidence that these studies are more likely to report findings supporting the efficacy and safety of the product than noncommercially funded studies.[12], [24], [25], [28], [29], [30] In our study sample, industry-funded studies were more likely to report an outcome favoring the use of the intervention than noncommercially-funded studies, and only 17% of drug studies and 26% of device studies used an active comparator. Research on drugs and devices would benefit from greater participation of non-stakeholders—such as government sponsors—as well as greater oversight in study design in order to ensure rigorous and valid assessments of the effectiveness of these treatments.[31]

There are several factors critical to ensuring the success of the new CE research initiative and the ability of CE research to improve clinical decision-making. Methodologically, CE studies must be large enough and have a sufficient patient follow up period to demonstrate not only equivalence, but superiority of one treatment compared to another.[32] Randomized controlled trials, which are typically designed to ascertain the efficacy of an intervention in select patient populations and tightly controlled settings, may not reflect real-world outcomes or be generalizable to routine clinical practice, which is one of the defining principles of CE research. By contrast, pragmatic clinical trials and observational studies may provide results that are directly pertinent to clinicians and patients choosing between available therapies.[33] In addition, comparative efficacy data must be timely and available prior to the widespread adoption of new products or interventions, as the lack of comparative evidence has resulted in the extensive use of a number of treatments later found to be less efficacious or safe than existing alternatives.[20], [31]

A limitation of our study is that outcomes data are not available for all studies since we chose to examine recent and ongoing studies, in order to ensure that our findings are most pertinent to the current state of CE research. However, it is unlikely that systematic bias produced our finding that CE studies are more likely to yield favorable outcomes. This finding is supported in prior literature and the sample of published results is of sufficient magnitude to demonstrate important and statistically significant differences in reported outcomes. [18], [22] We were not able to verify the accuracy of data provided by investigators, but information such as experimental treatment, comparator type, and funding source, are likely properly and reliably reported. Finally, there are some missing data in ClincialTrials.gov, including anticipated sample size for 1% of trials and study duration for 17% of trials.

In conclusion, less than a quarter of studies use an active comparator to measure the CE of the treatment under investigation. Based on outcomes reported in CE and non-CE studies, CE studies in general appear to provide more rigorous assessments of the interventions under study. Boosting noncommercial funding of CE studies may be particularly critical to drug and device studies in order to ensure unbiased data on how the intervention compares to other available treatments. Further study is necessary to understand the impact of CE research on healthcare reform and cost, as there is a risk when new treatments face a higher barrier to acceptance that some innovation may be slowed and development costs increased. On the other hand, we can expect that CE research will provide physicians and patients with substantially stronger evidence about which therapies are effective.

## Supporting Information

Table S1 **Study Topics and Keywords for Study Selection.** Clinical trials examined in the study pertained to these 15 research areas. The corresponding keywords were used to identify the trials in ClinicalTrials.gov using the embedded search function.
(DOC)
