# Supplementary material for: Comparative Effectiveness Research: An Empirical Study of Trials Registered in ClinicalTrials.gov
Source: PLoS One. 2012 Jan 9;7(1):e28820. doi: 10.1371/journal.pone.0028820 (PMC3253780; doi:10.1371/journal.pone.0028820)
Supplement: Table S1 — Study Topics and Keywords for Study Selection. Clinical trials examined in the study pertained to these 15 research areas. The corresponding keywords were used to identify the trials in ClinicalTrials.gov using the embedded search function. (DOC) [file pone.0028820.s001.doc]

| **Study Topic** | **Keywords for Study Selection^a^** | **References** |
| --- | --- | --- |
| Treatment of atrial fibrillation | atrial fibrillation |  |
| Treatment of hearing loss in children and adults | hearing loss  deafness  hearing impairment |  |
| Prevention of falls in older adults | falls |  |
| Treatment of gastroesophageal reflux disease | GERD  gastroesophageal reflux disease |  |
| Eradication of MRSA | MRSA  methicillin resistant staphylococcus aureus |  |
| Reduction of healthcare associated infections | healthcare associated infection  nosocomial infection  ventilator-associated pneumonia  catheter-associated urinary tract infection  catheter-related bloodstream infection  surgical site infection |  |
| Management strategies for localized prostatic neoplasms | localized prostate cancer  stage 1 prostate cancer  stage 2 prostate cancer  early stage prostate cancer |  |
| Treatment of low back pain | low back pain  lower back pain |  |
| Diagnosis and management of dementia | dementia |  |
| Treatment of dementia | dementia |  |
| Prevention and treatment of overweight and obesity in children and adolescents | overweight  obesity |  |
| Management of ductal carcinoma in situ | carcinoma in situ  ductal carcinoma in situ  DCIS  noninfiltrating intraductal carcinoma  intraductal carcinoma  ductal carcinoma in situ of the breast  localized breast cancer  stage 0 breast cancer |  |
| Prevention of dental caries in children | dental caries |  |
| Treatment of attention deficit/hyperactivity disorder in children | ADHD  attention deficit/hyperactivity disorder |  |
| Prevention of unintended pregnancies | contraception  unintended pregnancy  birth control  intrauterine device  condom  tubal ligation  tubal sterilization  family planning  cervical cap |  |

**Table S1. Study Topics and Keywords for Study Selection**

**^a^** Combinations of terms searched in addition to individual terms when multiple terms used.

**REFERENCES**

1. Safdar N, Abad C (2008) Educational interventions for prevention of healthcare-associated infection: a systematic review. Crit Care Med 36: 933-940.

2. Institute of Medicine (2009) Initial National Priorities for Comparative Effectiveness Research. Washington, DC: National Academies Press.

3. Thompson I, Thrasher JB, Aus G, Burnett AL, Canby-Hagino ED, et al. (2007) Guideline for the management of clinically localized prostate cancer: 2007 update. J Urol 177: 2106-2131.

4. Goodwin A, Parker S, Ghersi D, Wilcken N (2009) Post-operative radiotherapy for ductal carcinoma in situ of the breast. Cochrane Database Syst Rev: CD000563.

5. Virnig BA, Tuttle TM, Shamliyan T, Kane RL Ductal carcinoma in situ of the breast: a systematic review of incidence, treatment, and outcomes. J Natl Cancer Inst 102: 170-178.

6. Culwell KR, Curtis KM (2009) Use of contraceptive methods by women with current venous thrombosis on anticoagulant therapy: a systematic review. Contraception 80: 337-345.

7. Roberts A, Noyes J (2009) Contraception and women over 40 years of age: mixed-method systematic review. J Adv Nurs 65: 1155-1170.
